# FRESHER: Challenges and opportunities for building Health/Healthy futures

**DOI:** 10.1093/eurpub/ckac129.121

**Published:** 2022-10-25

**Authors:** G Giuffrè, S Giesecke

**Affiliations:** 1 ISINNOVA, Rome, Italy; 2 Austrian Institute of Technology GmbH, Vienna, Austria

## Abstract

**Background:**

FRESHER (2016-2018) Foresight and modelling for European health policy and regulation - is a H2020 project that aimed at representing alternative health Scenarios to test future policies for tacking Non-Communicable Diseases (NCDs). The key added-value consists in the combination of qualitative foresight and quantitative forecast approaches: a micro-simulation model, specifically developed by the project, computed the health outcomes of the four FRESHER Scenarios.

**Methods:**

The Scenarios building activities involved overall more than four-hundred experts and stakeholders, with different backgrounds within and beyond health fields, throughout all the steps of the process, in two surveys and nine workshops. The horizon scanning phase led to identify a wide range of societal trends that impact health and NCDs. Considering a long-term horizon at 2050, eight trends were then selected and ranked according to their importance and uncertainty.

**Results:**

In the Scenario Building phase, the combination of different evolutions of the selected trends resulted in four alternative futures: a Business as Usual Scenario, “The rich get healthier”, two response Scenarios, “We will Health you” and “Healthy together”, and a worst case Scenario “Desolation Health”. The scenarios imagine different futures and depict how health would subsequently change in such worlds.

**Conclusions:**

Given the increasing need to put health at the centre of all policies, it is necessary to make more evident the relationship between societal trends and their impact on health, specifically for NCDs. The translation of trends, and their evolutions, into changes of risk factors is a viable option to assess the impact of public health policy. FRESHER aimed to trigger a dialogue across different scientific and policy communities outside the health sector in order to define innovative strategies and identify disruptive innovations.

